# Genistein—Opportunities Related to an Interesting Molecule of Natural Origin

**DOI:** 10.3390/molecules27030815

**Published:** 2022-01-26

**Authors:** Ewa Garbiec, Judyta Cielecka-Piontek, Magdalena Kowalówka, Magdalena Hołubiec, Przemysław Zalewski

**Affiliations:** 1 Department of Pharmacognosy, Faculty of Pharmacy, Poznan University of Medical Sciences, 4 Święcickiego St., 60-780 Poznan, Poland; ewa.garbiec@student.ump.edu.pl (E.G.); pzalewski@ump.edu.pl (P.Z.); 2 Department of Bromatology, Faculty of Pharmacy, Poznan University of Medical Sciences, 42 Marcelińska St., 60-354 Poznan, Poland; mkowalowka@ump.edu.pl; 3 Department of Pediatric Gastroenterology and Metabolic Diseases, Poznan University of Medical Sciences, Szpitalna 27/33 St., 60-572 Poznan, Poland; magdalena.holubiec@ump.edu.pl

**Keywords:** genistein, functional food, soybean

## Abstract

Nowadays, increasingly more attention is being paid to a holistic approach to health, in which diet contributes to disease prevention. There is growing interest in functional food that not only provides basic nutrition but has also been demonstrated to be an opportunity for the prevention of disorders. A promising functional food is soybean, which is the richest source of the isoflavone, genistein. Genistein may be useful in the prevention and treatment of such disorders as psoriasis, cataracts, cystic fibrosis, non-alcoholic fatty liver disease and type 2 diabetes. However, achievable concentrations of genistein in humans are low, and the use of soybean as a functional food is not devoid of concerns, which are related to genistein’s potential side effects resulting from its estrogenic and goitrogenic effects.

## 1. Introduction

Genistein is an isoflavone, isolated for the first time in 1899, from belonging to the Fabaceae family *Genista tinctoria* L. [1]. Since then, its presence has been confirmed in many other plants of this family, such as *Medicago sativa* L. [2], *Lupinus albus* L. [3], *Apios americana* Medik [4] and *Pueraria montana* (Lour.) Merr [5]. Recently, genistein was, for the first time, reported in the marine algae *Padina tetrastromatica* Hauck [6]. Nevertheless, the basic source of isoflavones in the human diet still is *Glycine max* L. and soy products [7]. Of the total number of soy isoflavones, 60% is represented by genistein [8].

**Figure 1:**
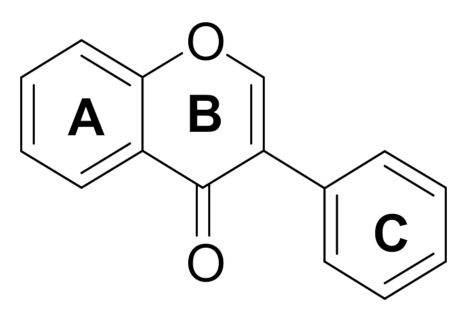
Chemical structure of 3-phenylchromen-4-one.

Genistein is a plant secondary metabolite from the isoflavone group. Its structure is based on the 3-phenylchromen-4-one configuration. It consists of two aromatic benzene rings (A and C) and one non-aromatic heterocyclic pyran ring (B) (Figure 1).

**Figure 2:**
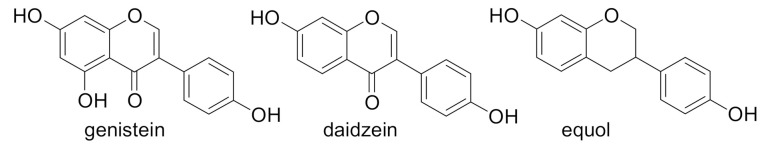
Chemical structure of genistein, daidzein and equol.

At the 5-, 7- and 4′- positions of phenylchromenone, there are hydroxyl groups. Genistein (4′,5,7-trihydroxyisoflavone) has one more hydroxyl (position 5) group compared to another plant secondary metabolite, daidzein (4′,7-dihydroxy iso flavone) (Figure 2).

**Figure 3:**
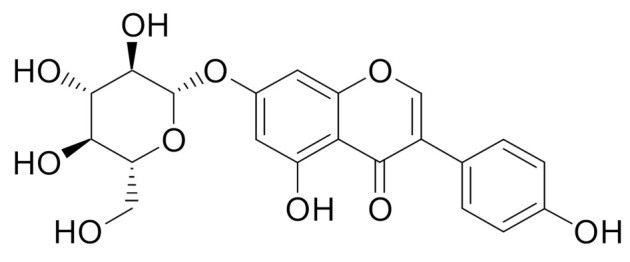
Chemical structure of genistin.

The majority of genistein occurs in plant raw materials in the glycoside form as genistin (genistein 7-glucoside) (Figure 3).

In the human body, the glucoside is converted into an active aglycone. Genistein can later be transformed into 5-hydroxy-equol by human intestinal bacterium *Slackia isoflavoniconvertens* [9], while one of the most important active metabolites of daidzein is equol (4′,7-isoflavandiol) (Figure 2) [10].

Genistein is found in soy products, legumes and many commonly consumed vegetables [11]. Studies published to date suggest that genistein represents a promising option for the prevention and treatment of many disorders, such as obesity [12,13], inflammatory bowel diseases [14,15] and neurodegenerative diseases [16,17,18]. It may have a protective effect against risk factors related to cardiovascular disease [19,20], exert a nephroprotective effect [21,22], delay ovarian aging [23] and promote wound healing [24]; it is a potentially promising compound for cancer chemoprevention [25,26] and the alleviation of postmenopausal symptoms [8]. Genistein consumed in soy products may affect the gut microbiome composition [27], which is considered to be associated with the host’s health [28].

As promising results are obtained in in vitro and animal studies, it is possible to suggest the usefulness of genistein in the prevention and treatment of some disorders whose case numbers are constantly increasing. These disorders include: psoriasis [29] and non-alcoholic fatty liver disease (NAFLD), with a 25.24% pooled global prevalence [30]; cataracts, one of the leading causes of vision impairment [31]; cystic fibrosis, which, although the survival has greatly improved, is still a serious, life-threating disease [32,33]; and diabetes, one of the top ten leading causes of death [34]. This review aims to present the results of studies on genistein’s action in those emerging research areas.

Since many disorders are related to lifestyle and diet, researchers are interested in the health benefits of genistein as the main polyphenolic compound of soy-based functional food.

## 2. Fermentation as a Way to Increase Genistein Content

It is well known that food can help prevent and cure diseases. Functional food is of particular importance in the prevention of civilization diseases. According to global trends, the amount of functional foods consumed increases significantly every year. On the other hand, detailed research is necessary to confirm the activity of the functional components in food.

Soybean products, especially, are an example of functional food. They contain a wealth of different ingredients, e.g., dietary fiber and crude protein, but probably one of the most valuable is genistein [35,36]. It is worth emphasizing that the content of genistein in soybeans is not influenced by the soil tillage system [37], while the technological processing of soybeans has a significant effect on this parameter. Okara (soybean waste from soymilk extraction) contains considerable amounts of isoflavones represented by genistein, with a content of 2.15 mg/100 g [38]. Okara is a valuable source of nutraceuticals with antioxidant, anti-inflammatory and enzyme-inhibiting properties, and the genistein contained in okara can be used as a functional food in pathological states based on oxidant or inflammatory processes as well as in the inhibition of overactive enzymes. It is also worth emphasizing that the concentration of genistein in fermented soybean products is relatively higher than both in soybeans and its unfermented products. When okara is fermented by *Rhizopus oligosporus*, the genistein concentration increases over two times in the obtained product [39].

An increase in the genistein content was also observed for fermented black soy beans by *Eurotium cristatum* YL-1 in solid-state fermentation [40]. Simultaneously, the bioavailability of isoflavones, especially genistein, significantly increases in fermented soybean products [41]. Another example of a functional food containing genistein is Cheonggukjang (CGJ). CGJ is a soybean paste fermented by *Bacillus*, *Lactobacillus*, *Leuconostoc* and *Enterococcus* strains. The fermentation process of CGJ increases the content of active substances, including genistein [42]. In soy milk fermented by *Schleiferilactobacillus harbinensis M1*, the glucoside form of isoflavone (genistin) was converted to the active aglycone form (genistein). The genistein concentration was 3.24-fold greater than in the unprocessed material. The functional properties of the thus obtained product were expanded [43]. A similar effect was observed for soy milk fermented with okara immobilized by *Lactobacillus plantarum* UFG10. The biotransformation of isoflavones from non-active to active took place [44]. *Bacillus subtilis* (BSNK-5) fermentation significantly increased the amount of functional components, such as genistein, in soymilk, but on the other hand, had a negative effect on the taste of the processed product [45]. Similar results were observed for soybean products prepared by other microorganisms (*Lactobacilli casei* and *Lactobacilli fermentum*). The concentration of genistein in the product obtained in such a manner was 2.65 mg/100 mL and, of course, was significantly higher than in the unfermented substrate. It was proven that a fermented soy drink completed with skim milk powder and whey protein concentrate could definitely be a nutritious beverage for people of all ages [46].

## 3. Genistein as a Biologically Active Plant Compound

### 3.1. Psoriasis

Psoriasis is a chronic disease with an inflammatory background. It mostly affects the skin and joints, but is considered a systemic disease because the ongoing inflammatory process affects the entire body. There are different types of psoriasis; the most common one, psoriasis vulgaris, which accounts for 90% of cases [47], is characterized by the occurrence of red plaques with silvery scales, typically located on the trunk, scalp and extremities [48]. The occurrence of skin lesions is associated with severe pruritus [49]. Psoriasis significantly reduces the quality of life [50]. It is prevalent all over the world and affects 2–3% of the population [51,52].

Psoriasis is a disease with a complex pathogenesis. At the molecular level, in the initial stage of the psoriatic process, an important role is played by dendric cells. When activated, among others, by tumor necrosis factor (TNF-α) and interferon-γ, the stimulated myeloid dendritic cells produce interleukins 12 and 23, which activate type 1 helper T cells (Th1) and type 17 helper T cells (Th17), respectively. Th1 and Th17 secrete TNF-α, interferon-γ and interleukins 17A, 17F and 22. These mediators cause the activation and proliferation of keratinocytes. The stimulation of keratinocytes leads to the production of such mediators as antimicrobial peptides, cytokines and chemokines, and it is accompanied by the infiltration of such cells as lymphocytes and monocytes as well as the development of inflammation [53,54,55,56].

It is also suggested that the onset and perpetuation of psoriatic inflammation may be associated with oxidative stress [57,58]. Therefore, it seems reasonable to take into account a balanced diet rich in antioxidants as an important tool in psoriasis management [59]. Potential benefits may also arise from including soy products in the diet [60], which are a source of the potent antioxidant, genistein [61].

The effects of genistein were examined in an in vitro model on a spontaneously immortalized human keratinocyte cell line (HaCaT) [62]. A reduction in the reactive oxygen species after the use of genistein in keratinocytes treated with TNF-α and lipopolysaccharide (LPS) was observed. It is possible to suggest that, regarding the ability of genistein to scavenge free radicals, this isoflavone may also inhibit the ROS/NF-κB pathway in HaCaT cells and thus reduce the production of inflammatory cytokines.

The impact of genistein on the gene expressions in skin biopsies and peripheral blood cells of psoriatic patients was investigated by Smolińska et al. [63]. The patients were treated with 75 mg and 150 mg genistein or received a placebo for 56 days. Genistein did not show high clinical efficacy in relation to such parameters as the Psoriasis Area and Severity Index, Physician Global Assessment or Body Surface Area. It was observed that genistein modulated the activity of psoriasis-related genes in skin biopsies and peripheral blood cells. Among the down-regulated transcripts were CCL4, IL8, NFKB1, STAT3, CXCL10 and IL6. IL1RN was stimulated in activity. Although an immune-mediated inflammatory background plays a crucial role in the development of psoriasis, genetic factors should also be considered [53].

Topical therapies are very often used in the treatment of psoriasis lesions [48]. A study, in which the effect of the topical application of an isoflavone extract on the skin was investigated, was carried out in mice [64]. The application of the isoflavone extract (10 mg/mL), prior to the application of imiquimod, decreased the development of erythema, lowered the trans-epidermal water loss and prevented epidermal hyperplasia. The isoflavone extract was also effective in reducing phosphorylation and the degradation of IκBα, and thus inhibited NFκB activation in normal human epidermal keratinocytes (NHEKs) stimulated by TNF-α and IL-17A. In another study, also involving an imiquimod-induced psoriasis-like skin model in mice, the effect of imiquimod application along with genistein (0.5% or 2%) was assessed [65]. Genistein was found to be effective in the alleviation of induced skin lesions, presumably because of its anti-inflammatory effects which result from the inhibition of NFκB and STAT3 signaling pathways.

Genistein could potentially be beneficial in the control of psoriasis when considering its potential to modulate genes involved in the inflammatory process. However, more research is needed to assess whether genistein is a good candidate for the treatment of inflammatory skin disorders.

### 3.2. Cataracts

A cataract is characterized as a progressive clouding of the lens leading to the deterioration of vision [66]. It mainly affects the geriatric population and is one of the main causes of blindness [67]. The leading factor in senile cataract development is the accumulation of free radicals and the resulting oxidative stress [66,67,68,69]. One of the most important risk factors, besides age, is diabetes [70]. Oxidative stress worsens and accelerates the formation of diabetic cataracts [71], but its development is mostly related to the polyol pathway; under hyperglycemic conditions, the accumulation of sorbitol, an intermediate product of the transformation of glucose into fructose catalyzed by the enzyme aldose reductase, is increased, leading to osmotic stress, cell lesions and lens damage [72].

The standard treatment for cataracts is surgery [73], and although there are ever more advanced methods [74], it is not devoid of complications [75]. Therefore, it is necessary to search for effective and safe natural substances to delay the progression of cataracts [76].

Patil et al. [77] assessed the anti-cataract activity of flavonoids against sugar-induced cataractogenesis using goat lenses. Lenses were incubated in Kreb Ringer Bicarbonate buffer with the addition of 30 mM glucose and 50 mM of each of the flavonoids. Genistein, along with chrysin, apigenin and baicalein, maintained the transparency and structural integrity of the lenses, as well as inhibited the formation of glycation mediated protein aggregates. Genistein exhibited a lower ability to inhibit aldose reductase compared to the abovementioned flavonoids.

In a study carried out by Kim et al. [78], genistein isolated from *Pueraria lobata* demonstrated both a dose-dependent inhibition of aldose reductase activity and significant improvement in the opacities of lenses. In high glucose-induced human lens epithelial cells (HLE-B3), treatment with genistein suppressed the expression of TGF-β2, αB-crystallin and fibronectin mRNAs.

The impact of genistein on cataracts induced by dietary galactose in rats was investigated by Huang et al. [79]. Rats were given genistein (15 mg/kg body weight), administered by oral gavage, daily for four weeks. Despite of the fact that genistein did not prevent the development of cataracts, it lowered the plasma glucose concentration, reduced the extent of the cataract and delayed its progression.

A result inconsistent with other studies was reported by Floyd et al. [80]. In this study, rats were fed ad libitum different diets containing genistein at a concentration of 0.018% in different forms: as an aglycone or β-glucoside (derived from Novasoy^®^200 and PRO-FAM 932 Soy Protein Isolate (SPI)), and also at a concentration of 0.2% as an aglycon. Genistein supplementation, independent of the genistein dose and form, accelerated the early onset of lens cataract formation in the male Ihara hereditary cataract rat (ICR/f rat). However, to assess the effect of genistein in a model of age-related cataract, this study used a hereditary cataractous rat. The action of genistein in this animal experimental model may be based on other mechanisms than in glycation-induced lens opacity.

Few studies have focused on the potential use of genistein in the management of posterior capsular opacification (PCO), one of the most common complications of cataract surgery [81]. It is a result of the proliferation, migration and accumulation of remnant lens epithelial cells in the posterior capsule, leading to effects on vision similar to cataracts [82]. Genistein depressed the growth of human lens epithelial cells (HLECs) in vitro. The inhibitory effect was increased by the use of a nanostructured lipid carrier (NLC) for genistein delivery [83,84]. In another study, nanostructured lipid carriers loaded with genistein were able to induce the apoptosis of HLECs. The anti-proliferation effect was enhanced by the chitosan modification of the surface of NLCs [85]. Recently, a genistein nanostructured lipid carrier, together with dexamethasone and moxifloxacin, was used in a multi-drug hydrogel to prevent the formation of PCO [86].

### 3.3. Cystic Fibrosis

Cystic fibrosis is a genetic, life-shortening disorder. A defect in the cystic fibrosis transmembrane conductance regulator (CFTR) protein that forms a chloride channel leads to the production of a thick and sticky mucus and impairment of the function of epithelial tissues [87]. Even though the main cause of mortality is changes in the respiratory system [88], the disease is systemic and also affects the function of other organs such as the pancreas, liver, intestine, kidneys and sweat glands [89]. Treatment is symptomatic and allows abatement of the irreversible changes, improving the quality of life [90].

In a rare S1045Y mutation in the CFTR gene, increased phosphorylation of tyrosine residues results in the ubiquitination and degradation of CFTR [91]. It is speculated that supplementation with genistein, as a tyrosine kinase inhibitor, may mitigate the symptoms of cystic fibrosis caused by S1045Y-CFTR [92].

The most common mutation, F508del, is associated with misfolding of the CFTR protein and its inability to reach the cell membrane [93]. Despite the fact that the genistein mechanism of action seems to be related mainly to controlling the opening of the CFTR chloride channel [91], in a study carried out by Lord et al. in which, over a period of 45 days, mice were fed ad libitum a diet containing genistein (600 mg/kg diet) or colyte-genistein, increased body weight of male F508del-CF mice and improved survival rate of F508del-CF female mice in the absence of laxatives were observed [94].

As a potentiator, genistein increases CFTR channel activity. In an ex vivo model, genistein and other polyphenol–curcumin, increased forskolin-induced swelling (FIS) of organoids, both in a dose dependent manner (effective concentration for genistein 3–100 μM and 25–200 μM for curcumin). Moreover, synergism of the action of the above-mentioned polyphenols with the ivacaftor was observed [95]. However, these results were not confirmed in clinical study. To evaluate in vitro findings, researchers performed three clinical trials. In a study number 1, patients received genistein in a dose between 3.3 and 5.0 mg/kg/day and curcumin in a dose between 102.9 and 138.5 mg/kg/day, three to four times a day for eight weeks. In a study number 2, patients were treated with ivacaftor in a dose of 150 mg, twice a day for eight weeks. In a study number 3, patients already treated with ivacaftor received genistein at a dose between 5.0 and 10.0 mg/kg/day for eight weeks, matched placebo for eight weeks, and went through a four weeks washout period [96]. Clinical parameters were improved after ivacaftor treatment. No statistically significant clinical effect was observed after supplementing genistein with curcumin or with ivacaftor, probably owing to their low plasma concentrations; analysis of the patients’ plasma showed that levels of curcumin were undetectable for most of samples. Median plasma concentrations for genistein were 0.01 μM in a study number 1 and 0.05 μM in a study number 3, which are lower than those levels determined by in vitro tests to be required for effective CFTR potentiation.

Despite promising results obtained in in vitro studies, use of genistein is limited because of its poor water solubility and low oral bioavailability followed by low plasma and tissue concentrations, variability of the gut microbiota, impact of the efflux transporters and metabolic enzyme activity [97,98].

### 3.4. Non-Alcoholic Fatty Liver Disease

Non-alcoholic fatty liver disease (NAFLD) is characterized by changes in the liver resembling those of alcohol abusers, but caused by other factors such as insulin resistance, diabetes [99], obesity or dyslipidemia. The increased accumulation of free fatty acids and triglycerides in the liver cells, followed by oxidative stress, lipid peroxidation and cytokine release, leads to the development of inflammation with associated fibrogenesis [100,101,102] and the development of non-alcoholic steatohepatitis (NASH) [103].

The prevalence of NAFLD is increasing worldwide [104,105], mostly because of a diet containing high fructose [106] and fat in addition to a sedentary lifestyle [102]. In terms of the differences between Asian and Western populations, the presence of NAFLD in the Western population is usually positively correlated with obesity and an elevated body mass index (BMI), while Asians develop NAFLD, despite a normal BMI [107,108], since they tend to have visceral obesity and related insulin resistance [105,109]. NAFLD can lead to cirrhosis, hepatocellular carcinoma and, ultimately, liver failure [110].

Although a key element in the treatment of NAFLD is lifestyle changes, which should include a healthy diet and physical activity [111], researchers are focused on new strategies and substances that can be helpful in NAFLD management. The collected information relates to publications published after 2018, in view of the fact that earlier works were described by Xin et al. [112].

Animal studies have shown that genistein can be effective in improving NAFLD. Promising results were obtained by Yin et al. [113] in their study, in which 12 weeks of a high fat and energy diet and a 10% sucrose solution added to drink resulted in destruction of the rats’ hepatic lobules, the accumulation of lipid droplets, increased levels of alanine aminotransferase (ALT) and aspartate aminotransferase (AST) and an increased expression of TLR4 compared to the low-fat fed group. In the high-dose genistein group (0.2% wt/wt), the hepatocytes were almost close to normal, liver injury was alleviated, the expression of TLR4 and level of TNF-α were decreased and inflammation was palliated.

Hens are appealing animals to model human NAFLD, and were used by Gao et al. [114] to investigate the comparative role of genistein and bisphenol A on NAFLD. Bisphenol A (50 μg/kg or 5000 μg/kg) aggravated pathological changes induced by a high-energy and low-protein diet. For 90 days, the hens were fed a diet supplemented with 40 mg/kg, 200 mg/kg or 400 mg/kg genistein. Genistein exposure, in a dose-dependent manner, decreased the ALT and AST levels, reduced the transcription levels of genes related to fatty acid uptake (LPL, Cd36) and fatty acid synthesis (ACC, FAS, SCD-1, SREBP-1) and, hence, inhibited the synthesis of fatty acids and reduced lipid accumulation. Genistein also improved insulin sensitivity and inhibited the NLRP3 inflammasome.

In another study carried out in mice, Zamani-Garmsiri et al. [115] combined metformin and genistein to study their effect in NAFLD. For 10 weeks mice were fed a high-fat diet, then treated for three months with metformin (2.3 g/kg diet) and genistein (2 g/kg diet) alone and in combination. Treatment with genistein and metformin alone as well as in combination decreased the body and liver weights of mice compared to those on the high-fat diet, lowered the levels of plasma insulin and blood glucose, decreased the levels of ALT and AST, reduced steatosis and reduced the expression of cytokines involved in the inflammatory process and the expression of PEPCK and G6Pase involved in gluconeogenesis genes. It was also observed that the combination of genistein and metformin reduced the expression of SREBP-1c, which plays a role in lipogenesis and the induced phosphorylation of GSK3.

The protective effect of genistein and 17β-estradiol on cells treated with free fatty acid was also studied in vitro [116]. The researchers used the human hepatoma cell line (Huh7.5 cells) and human primary hepatocytes treated with an NAFLD-like medium. Both phytoestrogen and estrogen reduced reactive oxygen species’ (ROS) production and increased the mitochondrial membrane potential, in addition to cell viability, in Huh7.5 cells and human primary hepatocytes. In the Huh7.5 cells, they also reduced the accumulation of triglycerides and attenuated the harmful effect of TNFα on cell viability.

The effect of genistein on the transcription factor involved in the catabolism (PPARα) and anabolism (SREBP-1c) of lipids was studied by Seidemann et al. [117]. After the incubation of primary human hepatocytes (PHHs) with free fatty acids and genistein, it was shown that the isoflavone in the concentration of 10 µM decreased the SREBP-1c protein expression in steatotic PHHs and exhibited agonistic activity in relation to PPARα in control PHHs; notwithstanding, this observation is inconsistent with results obtained by Pummoung et al. [118], and the role of PPARα in NAFLD is not fully elucidated.

The progressive form of NAFLD is NASH [103]. Witayavanitkul et al. [119] demonstrated that both genistein (at dose 16 mg/kg body weight, dissolved in 0.1% DMSO, administered by gavage daily for five weeks) and moderate running exercise mitigate the deleterious effects of a high fructose diet in ovariectomized rats with NASH. It is possible to suggest that this effect is associated with decreased MMP-12, IL-13 and HDAC3 protein expression. Analogous observation in relation to the protective role of genistein in NASH was produced in a previous study which was also performed on estrogen deficient rats with NASH [118]. Genistein (in the same dose of 16 mg/kg body weight, dissolved in 1 mL of 0.1% DMSO, given daily by oral gavage in an eight-week experiment) improved the histopathology results, reduced the MDA levels in ovariectomized rats with NASH, decreased the accumulation of hepatic fat and attenuated hepatocyte apoptosis. Gan et al. [120] studied the role of genistein in promoting miR-451 expression in hepatocytes to assuage NASH. In both in vivo (mice fed a diet supplemented with 100 mg/kg genistein for one month) and in vitro (20 μM genistein) studies, genistein reduced the expression of IL6 and TNFα and inflammation.

### 3.5. Type 2 Diabetes

The effect of genistein seems to be dependent on its bioavailability [121], which may be related to the increased consumption of soy products, following the observation that the increased soy consumption in Asians, compared to Westerners, is associated with a decreased incidence of breast cancer [26], osteoporosis [122], cardiovascular disease [123,124] and menopausal symptoms [8].

This hypothesis also seems to be confirmed by the fact that, due to changes in lifestyle and westernization of the Asian diet, an increase in the incidence of prostate cancer has been observed in recent years in Asian men, who previously had a lower incidence rate [125], and an increased risk of developing breast cancer is observed in immigrant Asian Americans [126].

Those findings were also studied in relation to type 2 diabetes, as, worldwide, there has been a dramatic increase in the number of cases of this chronic disease, with various complications affecting many organs. In 2013, the number of cases in Asia was already being described as an epidemic [127]. China has the largest diabetic population in the world [128,129]. Asians, despite having the same BMI as Westerners, have a higher risk of developing type 2 diabetes due to the tendency to accumulate visceral fat, which is associated with increased insulin resistance [130,131]. The main causes in the development of type 2 diabetes include genetic and lifestyle factors. Among other risk factors, a great role is played by dietary patterns [132,133].

For centuries, Asians have consumed large amounts of isoflavone-rich soybeans and soy products [134,135]. That has changed significantly with the shift towards a “western” diet rich in energy and fat [129,136], yet they still consume more soy products than Westerners [137]; that is why the influence of major soy isoflavones on the risks of developing type 2 diabetes is being investigated. Nevertheless, epidemiological studies conducted on the Asian population ended with various observations and conclusions.

In a study carried out on the Japanese population, the influence of the consumption of soy products on the risk of developing diabetes was examined. The dietary intake of genistein and daidzein was calculated based on tables assessing the isoflavone content in Japanese foods. No correlation was found between genistein and daidzein consumption and the risk of type 2 diabetes either in men or in women, but it turned out that a higher consumption of genistein and daidzein-rich foods may reduce the risk of diabetes in overweight women (and, to a lesser extent, also in postmenopausal women) [138]. Similarly, conclusions regarding the gender relationship were drawn by Ko et al. [139]. A high plasma genistein concentration (209 ng/mL) was related with a decreased risk of type 2 diabetes in Korean women. This correlation was found only in women who are known as equol producers. Equol is produced by intestinal bacteria [140,141] from daidzein, the most abundant, next to the genistein soy isoflavone [142]. The level of this metabolite in urine (0.33 mg/mol creatinine) was found to be associated with a decreased incidence of type 2 diabetes in a study carried out on the Chinese population [143]. Not only genistein but also other soybean isoflavones, particularly daidzein and its main metabolite equol, can be associated with anti-diabetic properties. However, this result is in contradiction with those obtained earlier in Chinese adults, when no correlation between the levels of genistein (1.17 nmol/mg creatinine), daidzein (1.43 nmol/mg creatinine) or equol (0.08 nmol/mg creatinine) in the urine and the risk of developing type 2 diabetes was confirmed [144]. Similarly, a study carried out by Ye et al. [145], in which participants received 10 g of soy protein supplemented with 50 mg of daidzein or 50 mg of genistein daily, did not confirm the effect of genistein or daidzein, after six months of supplementation, on insulin sensitivity or glycemic control in Chinese women.

In relation to Western populations, who have a much lower intake level of soy foods and isoflavones, the effect of genistein supplementation was studied in Caucasian postmenopausal women in Italy. One year of treatment with 54 mg of pure genistein daily in women with metabolic syndrome resulted in improvement in the parameters associated with a risk for diabetes [146]. An inverse association between the urinary excretion of genistein (122 nmol/g creatinine) and daidzein (311 nmol/g creatinine) and the risk of type 2 diabetes was also found in a study on women in the U.S. The effect was even stronger in postmenopausal women not taking hormone replacement therapy [147]. Another analysis of three U.S. cohorts showed that it was not tofu or soy milk consumption but rather isoflavone intake that was associated with a slightly decreased risk of type 2 diabetes in men and women [148].

It is clearly seen that the results are inconsistent. They differ not only between Asian and Western populations, but mixed results are also observed within the same populations. The authors of these studies point to various possible reasons for the heterogeneity of these findings, such as the difficulty in measuring the isoflavone intake in the diet—the qualitative and quantitative content of isoflavones is highly variable in various soy products and depends on the conditions of farming, processing, storage and subsequent preparation [139,147]. There is also a large difference in the content of isoflavones in soy products depending on the region [148]. Different findings may also result from the design of the study—prospective or nested control, different samples [143], sensitivity to measurement error owing to the dependence on the participant’s memory in studies based on a food frequency questionnaire [139,147], differences between the participants such as genetics, different cooking methods, preferences of different types of soy products [148] or differences in bioavailability [144,147]. Individual differences in the ability to metabolize isoflavones to active metabolites, in particular daidzein to equol, should especially be taken into account. Only 30–40% of the Western population is able to produce equol, in contrast to the Asian population, in which even 80% produces equol in the metabolism caused by gut bacteria [139,143]. In addition, soybean consumption varies greatly among the considered populations—it is much lower in Western populations [139]. It is possible that, in populations where soy products are not commonly consumed, isoflavones are more beneficial than in populations with a traditionally high consumption of soy products [143,144]. This effect appears to be specific to women, especially those in menopause, though the overall influence of gender on the considered differences remains unclear [145,149].

Despite the fact that the results obtained in human studies are inconsistent, promising results suggesting that genistein can affect glucose metabolism, counteract the effects of a high-fat diet and alleviate the complications of diabetes have been obtained in experimental animal studies. It has been shown that genistein can contribute to weight reduction, improvement of glucose metabolism [150] and the regeneration of damaged β-cells [151]. However, it still remains unclear whether genistein affects the plasma lipid profile. While some studies reported that genistein improves lipid metabolism [150] and, related to this improvement, prevents the accumulation of liver lipids [151], other studies did not confirm that genistein affects the plasma lipid profile [152]. The proposed mechanism of genistein action is therefore not the alteration of insulin sensitivity, but rather the enhancement β-cell function and the neutralization of free radicals [152], which are involved in the development of diabetes [153].

Animal studies also illustrate the importance of the role of genistein in counteracting the effects of a high-fat diet. Overweight is often associated with gut dysbiosis. Genistein may regulate the gut microbiota, resulting in intestinal barrier protection and a decrease in intestinal permeability [154], as a leaky gut is associated with the onset of diabetes [155]. The positive effect of gut microbiota altered by genistein on lipid and glucose metabolism was also observed in the offspring of high-fat diet dams [156].

As shown in animal studies, the potential applications for genistein also include the treatment of diabetes complications resulting from oxidative stress, the alleviation of neuropathic pain [157], the prevention and treatment of retinopathy [158] and the acceleration of wound healing in relation to the most feared complication of diabetes, the diabetic foot [159].

The animal studies in the above-mentioned disorders are summarized in Table 1. Animal studies provide a useful tool for determining the pathways of the beneficial effects of genistein, and their results are promising; nonetheless, they cannot be directly extrapolated to humans. Animal studies are also difficult to compare. They differ in the design of the experiment, with different methods for inducing the disorders and mimicking their symptoms, different dosages and modes of administration (gavage or mixing in food), differences in the form of genistein and its prior dissolution, variable durations of treatment, as well as the different animal species and their specific metabolic pathways. There is an urgent need for the development of new treatment strategies for the presented disorders. Genistein, although it appeared promising in the presented animal experiments, has uncertain relevance in humans. The effects of genistein in patients with the above-mentioned disorders are summarized in Table 2.

## 4. Limitations

The largest technological problem limiting the use of genistein as an ingredient of functional foods is its low water-solubility and poor oral bioavailability. The solution seems to be to carry out the procedure of encapsulating genistein in zein or zein/carboxymethyl/chitosan nanoparticles [160].

Another limitation of the clinical application of genistein is the possible side effects. The controversy over the consumption of soy and soy products rich in genistein is related to the estrogenic and goitrogenic effects [161].

Due to the 17β-estradiol structure similarity, genistein can bind to the estrogen receptor (with stronger affinity to ER-β than to ER-α) and is believed to mimic the effects of the body’s natural estrogen [162,163]. Despite this, the isoflavones in soy-based food and supplements do not appear to affect male fertility and do not cause feminization in men [164].

The effect of soy isoflavones on breast cancer associated with estrogens is unclear [165]. It is possible to suggest that elevated levels of estrogens are associated with an increased risk of breast cancer [166,167]. However, it seems that isoflavones can behave as estrogen agonists or antagonists and are considered as selective estrogen receptor modulators (SERMs) [168]. Their protective effect seems to be more pronounced in Asian women, probably because of the traditional consumption of higher amounts of less processed soy foods, followed by differences in gut microbiota and the bioavailability of isoflavones. A reduced risk of breast cancer appears to be affected by a higher consumption of soy during childhood [169,170,171].

It is also difficult to assess whether genistein affects thyroid function. This concern is raised owing to the fact that genistein has been proved to act as an inhibitor of thyroid peroxidase (TPO) in vitro [172] and shows such inhibition in rats [173]. Nevertheless, as follows from studies reviewed by Messina and Redmond [174], isoflavones do not appear to affect thyroid function in healthy adults. Possible adverse effects may be concomitant with iodine deficiency [175].

Safety concerns regarding the impact of the isoflavone on thyroid function are also related to soy-based infant formulas. Infant feeding consisting of soy-based formulas does not seem to affect later human development [176], and the iodine fortification of soy-based infant formula helps to prevent the occurrence of goiter in infants who receive high doses of phytoestrogens on such a diet [177]. Nonetheless, it may cause problems with controlling the disorder in children with congenital hypothyroidism [178].

Most of the raised concerns come from animal and in vitro data, while human studies do not seem to support these findings. Moreover, in view of the different metabolism of animals [179], the results cannot be directly extrapolated to humans.

## 5. Conclusions

Soybean, with one of its main compounds, genistein, is considered a health beneficial, functional food. The structural similarity of genistein and its metabolites to various types of cell receptors determines their multidirectional biological activity. Several of herein described in vitro and animal studies provide valuable information on the molecular basis of genistein’s action and show its potential usefulness in such disorders as psoriasis, cataracts, cystic fibrosis and non-alcoholic fatty liver disease. However, extrapolation of animal research data to humans is limited, as it is difficult to provide an appropriate animal model to mimic the human condition, especially considering the complexity of isoflavones’ metabolism and differences in bioavailability. Especially, the low oral bioavailability of genistein is a limitation in its clinical utility. It has to be taken into account that a wide range of the health benefits of soybean’s dietary intake is related to the content of isoflavones and active products of their metabolism. Therefore, it is always necessary to consider population variability when studying its action in specific diseases.

The biological activity of genistein compounds will increase the demand for the plant’s raw material, soybean. Soybean should therefore not only be considered as a starting material for the production of genistein, but also as a functional food component that meets the criterion.

## Figures and Tables

**Table 1:** Effects of genistein in in vivo models of selected disorders.

Disease	Models	Period	Genistein Dosage and Administration	Plasma Concentration of Genistein	Main Results	Year Published	Reference
Cataracts	Cataract induced by galactose-rich diet in male Long–Evans rats	4 weeks	15 mg/kg body weight oral gavage	N/A	1. Genistein treatment did not prevent cataract formation 2. Score and extent of cataracts↓ 3. Serum glucose↓ 4. Serum testosterone↑	2007	[79]
Cataracts	Male hereditary cataractous ICR/f rat	85 days	0.018% and 0.2% in the diet (as an aglycone or β-glucoside)	0.018% Genistein: 113.4 ± 63.2 nmol/L 0.20% Genistein: 2138.1 ± 720.8 nmol/L NovaSoy^®^200: 120.9 ± 107.3 nmol/L SPI: 72.5 ± 38.9 nmol/L	1. Acceleration of early stages of cataractogenesis	2011	[80]
Cystic fibrosis	F508del-CF male and female mouse	45 days	600 mg genistein/kg in the diet	N/A	1. Female survival rate↑ 2. Male body weight↑ 3. SGLT-1 expression↑	2018	[94]
NAFLD	High-fat high-sucrose diet-fed SPF male Sprague-Dawley rats	12 weeks	0.1% and 0.2% in the diet	N/A	1. NAFLD activity score↓ 2. Body weight↑, liver index↓ 3. Liver and serum TG↓, TC↓; LDL-C, HDL-C had no difference 4. ALT↓, AST↓ 5. Blood glucose did not change significantly; insulin↓, HOMA-IR↓ 6. Liver TNFα↓, serum endotoxin↓, TLR4↓	2019	[113]
NAFLD	High-energy and low-protein diet-fed Hy-Line Brown laying hens	90 days	40 mg/kg, 200 mg/kg, 400 mg/kg in the diet	N/A	1. Hepatic steatosis↓, NAFLD activity score↓ 2. ALT↓, AST↓ 3. Serum TG↓, TC↓, LDL-C↓, HDL-C↑ 4. Expression of AMPKα↑, CPT-1↑, PPARα↑ mRNA levels; ACC↓, FAS↓, SCD-1↓, SREBP-1↓, LPL↓ and Cd36↓ 5. Restored insulin sensitivity 6. mRNA and protein levels of NLRP3↓, caspase-1↓, IL-18↓, IL-1β↓ 7. Up-regulation of ERα in dose-dependent effect, no changes in ERβ expression	2021	[114]
NAFLD	High-fat diet-fed male C57BL/6 mice	3 months	0.2% in the diet (alone and in combination with metformin)	N/A	1. Body and liver weight↓ 2. Blood glucose↓, plasma insulin↓, HOMA-IR↓; ameliorated glucose tolerance 3. ALT↓, AST↓, liver and plasma TG↓; reduced steatosis 4. Expression of FAS↓, CPT1↑, SREBP-1c↓ (combination of metformin and genistein) 5. NAFLD activity score↓ 6. Liver phospho-AMPK protein level↑, TNF-α↓, IL-1β↓, IL-6↓, NF-κB p65 protein level↓ 7. Hepatic expression level of PEPCK↓ and G6Pase↓; hepatic GSK-3β phosphorylation↑	2020	[115]
NAFLD	High-fat high-fructose diet-fed ovariectomized female Sprague-Dawley rats	5 weeks	16 mg/kg body weight dissolved in 0.1% dimethyl sulfoxide oral gavage	N/A	1. Body weight did not change significantly 2. Hepatic MMP-12 expression↓, level of HDAC3 expression↓, IL-13↓ 3. NASH activity score↓	2021	[119]
NAFLD	High-fat high-fructose diet-fed ovariectomized female Sprague-Dawley rats	8 weeks	16 mg/kg body weight dissolved in 1 mL 0.1% dimethyl sulfoxide oral gavage	N/A	1. Improved liver steatosis 2. Hepatic MDA level↓ 3. Hepatic TG↓, FFA↓ 4. PPARγ↓, adiponectin↑	2021	[118]
NAFLD	High-fat diet-fed lipopolysaccharide-injected female ICR mice	1 month	100 mg/kg in the diet	N/A	1. Liver weight index↓ 2. Hepatic TNFα↓, IL6↓, IL1β↓ 3. Expression of miR-451↑, Cab39↓ 4. NASH activity score↓	2019	[120]
Diabetes	High-fat diet-fed C57BL/6J female mice	8 weeks	2 g/kg diet	N/A	1. Body weight↓ 2. Blood glucose levels↓, serum insulin level did not change significantly, HOMA-IR↓ 3. Serum TG↓, LDL-C↓, FFA↓; TC and HDL-C did not change significantly 4. Up-regulated genes: Per1, c-Fos, Calm1, Gng5 5. Down-regulated genes: Grin1, Cacna1g, Kir3.1, Adcy4, Gucy1a2	2019	[150]
Diabetes	High-fat diet-fed streptozotocin-injected female Wistar rats	4 weeks	10 mg/kg and 20 mg/kg in the diet	N/A	1. Blood glucose↓, serum insulin level↑, HOMA-IR↓ 2. Cholesterol↓, triglyceride↓, LDL↓, VLDL↓, HDL↑ 3. Regeneration of β-cells	2020	[151]
Diabetes	High-fat diet-fed streptozotocin-injected male C57BL/6 mice	8 weeks	250 mg/kg diet	N/A	1. Body weight, major organ weight did not change significantly 2. Fasting blood glucose↓ 3. TC, TG, HDL-C did not change significantly 4. β-cell mass improved	2012	[152]

**Table 2:** Effects of genistein in patients with selected disorders.

Disease	Participants/ Type	Period	Genistein Dosage	Concentration of Genistein	Main Results	Year Published	Reference
Psoriasis	34/randomized, double-blind, placebo-controlled trial	56 days	75 mg and 150 mg	N/A	1. Up-regulated genes: IL1RN 2. Down-regulated genes: CCL4, NFKB1, STAT3, CXCL10, IL6, IL8	2019	[63]
Cystic fibrosis	Study 1: 13 Study 3: 14/multicenter clinical trials	8 weeks	Study 1. 3.3 and 5.0 mg/kg/day + curcumin in 3–4 doses Study 2. ivacaftor Study 3. 5.0 and 10.0 mg/kg/day + ivacaftor	Study 1. 3 µg/L (0.01 µM) Study 3. 14 µg/L (0.05 µM)	1. Study 1, 3: no statistically significant clinical effect	2020	[96]
Diabetes	120 postmenopausal women/randomized, double-blind, placebo-controlled trial	1 year	54 mg	790 ± 95 nmol/L (serum)	1. HOMA-IR↓, blood glucose↓, insulin↓ 2. TC↓, TG↓, HDL-C↑ 3. Serum adiponectin↑, visfatin↓, homocysteine↓	2013	[146]
Diabetes	165 women/randomized, double-blind, placebo-controlled trial	24 weeks	10 g of soy protein plus (i) no addition, (ii) 50 mg of daidzein, (iii) 50 mg of genistein	16.6 mmol/24 h (urine)	1. Glycemic control, insulin resistance-no significant improvement	2014	[145]
